# Depression Awareness and Self-Management Through the Internet: Protocol for an Internationally Standardized Approach

**DOI:** 10.2196/resprot.4358

**Published:** 2015-08-06

**Authors:** Ella Arensman, Nicole Koburger, Celine Larkin, Gillian Karwig, Claire Coffey, Margaret Maxwell, Fiona Harris, Christine Rummel-Kluge, Chantal van Audenhove, Merike Sisask, Anna Alexandrova-Karamanova, Victor Perez, György Purebl, Annabel Cebria, Diego Palao, Susana Costa, Lauraliisa Mark, Mónika Ditta Tóth, Marieta Gecheva, Angela Ibelshäuser, Ricardo Gusmão, Ulrich Hegerl

**Affiliations:** ^1^ National Suicide Research Foundation Cork Ireland; ^2^ Department of Epidemiology and Public Health University College Cork Cork Ireland; ^3^ University Hospital Department of Psychiatry and Psychotherapy University of Leipzig Leipzig Germany; ^4^ German Depression Foundation Depression Research Centre Leipzig Germany; ^5^ Nursing, Midwifery & Allied Health Professions Research Unit School of Health Sciences University of Stirling Stirling United Kingdom; ^6^ LUCAS Centre for Care Research and Consultancy University of Leuven Leuven Belgium; ^7^ Estonian-Swedish Mental Health and Suicidology Institute Tallinn Estonia; ^8^ Institute of Social Work Tallinn University Tallinn Estonia; ^9^ Health Psychology Research Center Sofia Bulgaria; ^10^ Institute for Population and Human Studies Bulgarian Academy of Sciences Sofia Bulgaria; ^11^ Institut de Neuropsiquiatria i Addicions, Hospital del Mar CIBERSAM Universitat Autònoma de Barcelona Barcelona Spain; ^12^ Universitat Autònoma de Barcelona Department of Psychiatry and Forensic Medicine Barcelona Spain; ^13^ Centro de Investigación Biomédica Red de Salud Mental CIBERSAM Barcelona Spain; ^14^ Institute of Behavioural Sciences Semmelweis University Budapest Hungary; ^15^ Corporació Sanitària Parc Taulí de Sabadell Department of Mental Health Sabadell Spain; ^16^ NOVA Medical School Universidade Nova de Lisboa Lisbon Portugal; ^17^ Family National Association Sofia Bulgaria; ^18^ pro mente Tirol Innsbruck Austria; ^19^ Instituto de Saude Publica Universidade do Porto Porto Portugal

**Keywords:** cognitive behavioral therapy, computerized, depression, Internet-based, primary care, self-management

## Abstract

**Background:**

Depression incurs significant morbidity and confers increased risk of suicide. Many individuals experiencing depression remain untreated due to systemic and personal barriers to care. Guided Internet-based psychotherapeutic programs represent a promising means of overcoming such barriers and increasing the capacity for self-management of depression. However, existing programs tend to be available only in English and can be expensive to access. Furthermore, despite evidence of the effectiveness of a number of Internet-based programs, there is limited evidence regarding both the acceptability of such programs and feasibility of their use, for users and health care professionals.

**Objective:**

This paper will present the protocol for the development, implementation, and evaluation of the iFightDepression tool, an Internet-based self-management tool. This is a cost-free, multilingual, guided, self-management program for mild to moderate depression cases.

**Methods:**

The Preventing Depression and Improving Awareness through Networking in the European Union consortium undertook a comprehensive systematic review of the available evidence regarding computerized cognitive behavior therapy in addition to a consensus process involving mental health experts and service users to inform the development of the iFightDepression tool. The tool was implemented and evaluated for acceptability and feasibility of its use in a pilot phase in 5 European regions, with recruitment of users occurring through general practitioners and health care professionals who participated in a standardized training program.

**Results:**

Targeting mild to moderate depression, the iFightDepression tool is based on cognitive behavioral therapy and addresses behavioral activation (monitoring and planning daily activities), cognitive restructuring (identifying and challenging unhelpful thoughts), sleep regulation, mood monitoring, and healthy lifestyle habits. There is also a tailored version of the tool for young people, incorporating less formal language and additional age-appropriate modules on relationships and social anxiety. The tool is accompanied by a 3-hour training intervention for health care professionals.

**Conclusions:**

It is intended that the iFightDepression tool and associated training for health care professionals will represent a valuable resource for the management of depression that will complement existing resources for health care professionals. It is also intended that the iFightDepression tool and training will represent an additional resource within a multifaceted approach to improving the care of depression and preventing suicidal behavior in Europe.

## Introduction

### Overview

Depression and suicidal behavior, including both suicide and nonfatal self-harm, are 2 important and largely overlapping public health problems in Europe [1]. European countries are overrepresented among the highest national rates of suicide in the world [2,3], and unipolar depressive disorders are the third cause of disability-adjusted life years in Europe [2]. People suffering from major depression are 21 times more likely to die by suicide than nondepressed individuals [4]. Depressive disorders are present in approximately half of completed suicides [1,5], and this proportion is even higher if the presence of subclinical depressive symptoms is considered [6,7].

### Research Context of iFightDepression: Depression and the Current Situation of Care

Given the connection between depression and suicide, it is not surprising that improving the care of people with depression is considered an effective suicide prevention approach [8]. Several successful European studies provide support for this approach. The pioneering Gotland study [9,10], the Nuremberg Alliance against Depression [11,12], and further studies evaluating multilevel community-based interventions, such as the implementation of a local Alliance against Depression in Hungary [13], have demonstrated that interventions to improve the recognition and treatment of depression can effectively reduce the incidence of suicidal acts. The European Alliance Against Depression (EAAD) [14] and the European Commission-funded “Optimising Suicide Prevention Programs and their Implementation in Europe (OSPI-Europe)” project [15] have explored the potential of such community-based interventions to improve awareness of depression and to prevent suicidal behavior across several European countries. These interventions operate on multiple levels within the community, including the following: (1) cooperation with primary care services, focusing on trainings for general practitioners (GPs) to improve professional recognition of depression, including education about lethal medication and information regarding the detection, assessment, and diagnosis of depression; (2) public relation activities involving education of the broad public with a multifaceted depression awareness campaign; (3) cooperation with community facilitators and stakeholders, including training workshops focusing on recognition of depression, facilitation of access to appropriate care, and cooperation to restrict access to lethal means; and (4) facilitation of care and support for patients, high-risk groups, and their relatives, with the provision of information regarding helplines and emergency contacts and the initiation of, and support for, self-help groups [16].

Such multilevel interventions have demonstrated effectiveness with regard to the reduction of stigma toward depression, improvement of both lay and professional knowledge and awareness of depression, and increased motivation of individuals to seek help for depression as a result of broad general public health campaigns and increased professional recognition of depression. However, despite this promising evidence, the need to improve the care for individuals who are motivated to seek help for their depression has become evident [17,18]. Specifically, as the number of depressed individuals motivated to seek help increases, the demand on available resources and support services increases as well. As a result, individuals may encounter structural barriers such as limited availability of specialized care in rural areas, or lengthy waiting times for psychotherapeutic treatment [19]. Thus, once a person decides to access help for depression, or professional education increases awareness and detection of depression in clinical practice, there may be limited effective assistance available.

The importance of improving the care for individuals with depression is also demonstrated in light of diagnostic and therapeutic deficits at the primary care level. Patients with depression who seek help often present to general practice with mainly somatic complaints [20,21]. If depression occurs in individuals living in difficult life circumstances (eg, those experiencing somatic comorbidities and unemployment), it is often seen as a secondary phenomenon, a reaction to life circumstances, and not as an independent severe disorder that should be treated according to appropriate guidelines. In addition, recent studies have demonstrated that depression is underdetected and inadequately screened within primary care [22,23]. These are a number of reasons why only approximately 50% of depressed patients are correctly diagnosed at the primary care level [24,25].

Even if a diagnosis is made, very often specific psychotherapy is not available, nor is pharmacotherapy prescribed. When pharmacotherapy is initiated, there are sometimes challenges with drug dosage and time span [26,27]. Finally, even if pharmacological or psychological treatment is offered, there may be considerable compliance problems [28,29]. Moreover, many national health services in Europe are increasingly ill placed to provide specialized interventions for depression in light of the current economic recession: governmental cost-saving measures adopted in several countries have included the reduction of budgets for mental health services with subsequent effects on service availability [30]. Given the decreasing availability of effective treatment services for depression, it is apparent that additional resources are urgently needed to offer support to both patients and health care professionals for the management of depression.

### Depression and Self-Management Using the Internet

Because of the current constraints within national health services and the resultant limitations on delivering best practices of mental health care delivery within primary care [31], much of the responsibility for the initial care of mild to moderate depression lies with primary care providers. In such settings, there is a need for treatment complementarity: primary care providers and patients should be provided with *a range* of evidence-based and effective options for the management of depression. Antidepressants are effective and are widely used to treat depression, but patients may be reluctant to use antidepressant medication. Clinical guidelines regarding the management of depression now recommend a “stepped-care” approach to depression, whereby lower intensity psychosocial interventions may be used to treat lower levels of depression [32]. This is important, given that even mild or minor forms of depression negatively affect quality of life [33], and are associated with functional impairments [34], increased mortality, and risk of transition to severe depression and suicidal behavior [35]. Lower intensity psychosocial interventions often incorporate the concept of self-management, an approach that can complement treatment combinations for mild and moderate depression by empowering patients while reducing demands on health care services [36].

Self-management is an important aspect in the management of long-term illnesses. It refers to “interventions, trainings, and skills by which patients with a chronic condition, disability, or disease can effectively learn how to take care of themselves and effectively deal with difficult situations” [37]. Originally applied to chronic somatic diseases with success [38-40], it is increasingly being applied to mental health [41]. The Internet has provided new avenues for self-management as it enables cost-effective access to self-management resources at the patient’s own convenience and in a location of their choice. Computerized cognitive behavioral therapy (cCBT) is one type of a lower intensity intervention recommended for the treatment of mild to moderate depression in several clinical guidelines [32,42], which incorporates the principles of self-management.

### The Preventing Depression and Improving Awareness Through Networking in the European Union Project

The Preventing Depression and Improving Awareness through Networking in the European Union (PREDI-NU) is an international European Union-funded project that involves expert clinicians and researchers in the fields of depression and suicide prevention from 11 European countries, in addition to an international expert advisory panel. The project was funded from September 2011 to September 2014 and builds upon the aforementioned research by the EAAD and OSPI-Europe. Specifically, the PREDI-NU project intends to fill gaps in the availability of evidence-based self-management resources for mild to moderate depression through information and communications technology. In light of this, it encompasses the following 3 main aims:

1. The development of a multilingual European depression awareness and information website [43], to raise awareness of depression and suicidal behavior, to improve knowledge and attitudes regarding depression and suicidal behavior, and to promote help seeking and mental health.

2. The development of an evidence-based, multilingual self-management program for mild to moderate depression to be implemented and “guided” by primary care practitioners or mental health professionals who attend standardized professional training.

3. Implementation of the self-management program in 5 European regions, in addition to evaluation of the acceptability of the program and feasibility of its use, to inform future implementation of the program after project running time.

The purpose of this paper is to describe the study protocol regarding the development, implementation, and evaluation of the self-management program.

## Methods

### Development of the Self-Management Program

A systematic review informed by the realist approach [44] explored the evidence for cCBT. This was conducted during the 1st year of the PREDI-NU project to inform development of the self-management program. The systematic review aimed to specifically examine (1) what interventions work, for whom, and in what circumstances, and (2) to identify best practice recommendations for implementation of self-help ehealth technologies. This review consisted of a rigorous systematic literature search resulting in 52 papers, of which 22 were meta-reviews or systematic reviews, 5 were guidelines, and the rest were feasibility studies or studies informing the development, implementation, or use of cCBT. For the purposes of this protocol paper, results from the review will be referred to generally, and extensive results will be published in a separate future study.

The review indicated that numerous cCBT programs for depression have been developed, and that positive randomized controlled trial evidence exists for several packages, namely, Beating the Blues [45], MoodGYM [46,47], and Colour Your Life [48]. However, there is no clear evidence of any one program being more effective than another; additionally, there is little knowledge to guide the development or implementation of such interventions. Furthermore, despite evidence of their effectiveness, there is limited evidence on the acceptability (to both patients and professionals) and feasibility of the use of Internet-based self-management interventions for the management of depression, which may limit their uptake in primary care practice [49] and is likely to be a contributing factor to the high rates of attrition and noncompletion of such programs. Moreover, many established programs are available only in English and only with payment of a fee to the user or for the general practice. Although the review indicated that guided Web-based interventions are more effective in reducing depression than unguided programs [32,50-52], there is no clear evidence regarding the optimal level or format of delivery of guidance, and little consistent evidence to support implementation of cCBT overall.

This systematic review was supplemented by scoping existing cCBT websites internationally and identifying key features for inclusion within the self-management program to be developed. To ensure that procedures and materials meet international standards of evidence-based practice, a rigorous consensus process informed development of the program, involving a panel of international experts on cCBT and a scientific advisory board of international experts with extensive experience of Web-based interventions for depression and related mental health issues. Representatives from patient and family organizations also provided input into this consensus process.

### Design and Contents of the Self-Management Program

**Figure 1:**
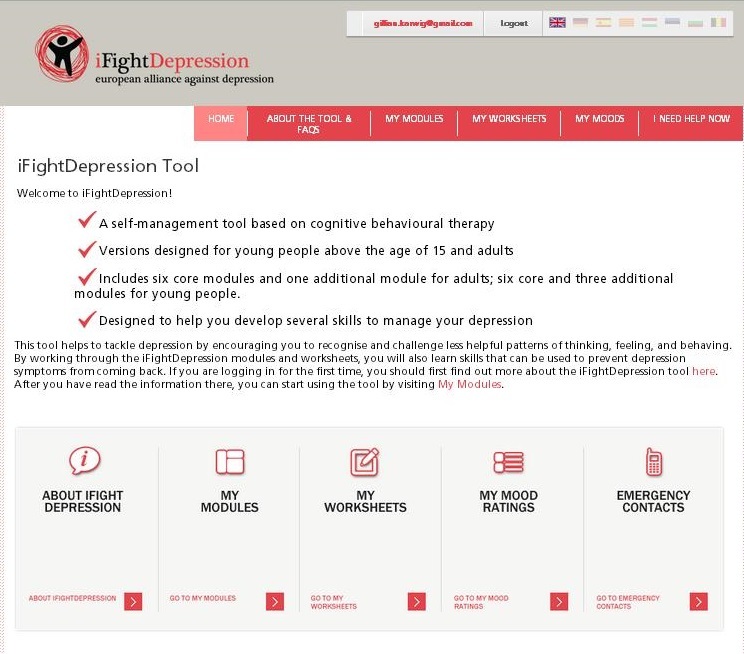
Home page of the iFightDepression tool.

Using the aforementioned, evidence-based, and best-practice approach, the PREDI-NU consortium developed the iFightDepression tool, a guided Internet-based self-management program for individuals experiencing mild to moderate depression, with versions for both adults aged 25 years and older and young people between 15 and 24 years of age. The iFightDepression tool is derived from a cognitive behavioral therapy approach and primarily focuses on the associations between thoughts, feelings, and behavior. A screenshot of the home page of the iFightDepression tool is shown in Figure 1.

In addition to introductory and emergency contact material, the iFightDepression tool comprises 6 core modules relating to behavioral activation, sleep and mood monitoring, and cognitive restructuring: “Thinking, Feeling, and Doing”; “Sleep and Depression”; “Planning and Doing Things That You Enjoy”; “Getting Things Done”; “Identifying Negative Thoughts”; and “Changing Negative Thoughts”. Individuals are encouraged to complete the modules in the structured order in which they appear in the tool; this is to encourage individuals to initiate behavior activation, to examine the relationship between their sleep, moods, and activities, and to integrate positive activities into their daily schedules before the modules relating to cognitive restructuring are undertaken, as these may be more challenging. It has also been suggested that individuals complete the modules at a rate of no more than 1 module/week, with an estimation of 30-40 minutes for the completion of each module. However, while these instructions are recommended, users can determine their personal pace and order of modules if they wish, as suggested by patient representatives involved in the development of the tool. Each module incorporates associated tasks and corresponding worksheets to consolidate learning and promote self-monitoring. In addition to encouraging users to plan and reflect on activities, moods, and thoughts, the tasks and worksheets help users to observe the associations between what they think, what they do, and how they feel.

The “Sleep and Depression” module is innovative and is based on the recently published vigilance regulation model of affective disorders [53]. It supports patients to examine the relationship between the duration of their sleep/time in bed and mood, and to identify personal optimal sleep times. Research suggests that there is a subgroup of patients who feel more tired, exhausted, and depressed after longer-than-usual sleep/time in bed, and that they show improvement after shortening of sleep/time in bed [53]. The effects of partial or total therapeutic sleep deprivation on depression are striking and well established [53]. However, in contrast to chronic sleep restriction, therapeutic sleep deprivation cannot easily be implemented in routine care or self-management approaches. The iFightDepression tool encourages patients to explore the association between their sleep patterns and their mood and to adjust their personal sleeping habits accordingly.

In addition to the 6 core modules, there are optional modules (2 tailored specifically for young people and 1 for both young people and adults) that address related psychosocial issues, namely, relationships, social anxiety, and healthy lifestyle habits.

The iFightDepression tool also encourages individuals to monitor their mood using an embedded, electronic version of the Patient Health Questionnaire-9 (PHQ-9) [54], a short questionnaire that measures the presence/absence of depressive symptoms in the 2 weeks prior to completing the questionnaire, in addition to the frequency of these symptoms. Individuals’ scores on the PHQ-9 are automatically plotted on a graph, which allows individuals to visually and clearly track their mood over time. The PHQ-9 was also included within the tool as a safety measure—should an individual’s depressive symptoms worsen (as evidenced by 3 consecutive scores reflecting a specific result of “severe depression,” and/or a positive response to the 9th item within the questionnaire—“thoughts that you would be better off dead or of hurting yourself in some way”), a feedback window automatically appears that encourages the user to contact their GP, health care professional, or emergency services.

### Implementation of the iFightDepression Tool

#### Specific Protocol Regarding Implementation of the Tool

The iFightDepression tool was implemented through GPs and health care professionals who completed a standardized 3-hour training workshop regarding implementation and guidance of the tool. Specifically, the tool was targeted toward professionals working in the area of mental health who are experienced in the assessment and diagnosis of depression; for example, GPs, family physicians, psychologists, psychiatrists, community mental health nurses, mental health social workers, and clinical nurse specialists. Trained health care professionals were instructed to initially assess patients presenting with depression for eligibility to use the tool (ie, a diagnosis of mild to moderate depression); it was recommended to professionals to use the PHQ-9 or the WHO-Five Well-Being Index in addition to their clinical judgment to ensure that the iFightDepression tool represented an appropriate resource for a patient, given his/her current level of depression. Professionals subsequently provide guidance as the individual commences use of and progresses through the tool.

The iFightDepression tool is intended to complement the available approaches for clinicians regarding the management and treatment of depression, as an adjunct to a patient’s usual care. However, the iFightDepression tool is also intended to be used as a single resource for an individual when deemed appropriate; for example, to bridge waiting times for patient access to face-to-face psychotherapy.

#### Implementation Phases of PREDI-NU

Two phases of implementation occurred during PREDI-NU, with a pilot phase undertaken at the beginning of the 2nd year of PREDI-NU in 5 European regions, followed by a first-phase evaluation to inform enhancement of the iFightDepression tool for continued implementation in these regions. Specifically, following the pilot phase, feedback about the acceptability of the tool and feasibility of its use from patients, health care professionals, and a group of healthy Internet users, in addition to recommendations and input from the scientific advisory board of international experts, was used to enhance all materials relevant to the intervention, including the tool itself and materials for the professional training workshop. The 3rd and final year of the project involved the implementation of the optimized tool, aiming at sustainable implementation through the development of materials for Train-the-Trainer workshops to qualify senior health professionals to deliver the standardized 3-hour professional training workshops to peers, colleagues, and additional interested professionals.

Before the pilot phase of implementation, local advisory panels were formed in each of the intervention regions, allowing the regions to explore and balance adaptation to local resources and constraints and facilitate access to health care professionals. Shared decision making was undertaken across regions regarding adaptation to procedures of implementation to ensure what could be described as an “empowerment implementation” approach [55].

#### Professional Guidance

“Guidance” was incorporated into the protocol for implementation of the iFightDepression tool as a key element, whereby individuals both maintain contact with and receive support from a trained GP or health care professional throughout their use of the tool. Guidance was included for the following reasons:

1. The systematic review of previous cCBT interventions demonstrated that guided Internet-based interventions are more effective than nonguided interventions.

2. It is expected that the incorporation of guidance may minimize potential attrition of individuals using the iFightDepression tool, as the review indicated that some level of human contact may improve completion rates of online self-help interventions by increasing motivation.

3. Furthermore, the inclusion of guidance represents an additional safety net as individuals whose depressive symptoms worsen throughout their use of the tool will be encouraged to contact and inform their GP or mental health professional: both during interaction with their health care professional and by way of the informative “feedback” window that is displayed within the tool if patients demonstrate more severe depressive symptoms or suicidal and self-harm ideation after completing the PHQ-9.

The systematic review also indicated that no clear evidence exists regarding the optimal level or format of delivery of guidance. A standardized set of guidelines was thus established regarding guidance of the iFightDepression tool. It comprised the following:

1. Guidance would amount to at least 45 minutes over the course of an individual’s use of the iFightDepression tool, and that guidance would be mainly motivational in nature.

2. The nature of the guidance can be flexible and may differ between professional groups (GPs, psychotherapists, other mental health professionals) as they are working within different settings and time constraints. The exact means of implementation may also depend on the personal preference and working style of the professional.

3. There should be at least two face-to-face sessions in addition to the initial personal appointment where the tool is recommended to patients: halfway through a patient’s use of the tool and upon completion of the tool. These face-to-face sessions may be incorporated within standard follow-up appointments provided by the health care professional as part of treatment as usual. This level of guidance is in line with previous studies and national guidelines for the primary care of depression [32].

4. Additional guidance can be provided by telephone; however, it may also be provided in other ways, such as by email or text.

#### Professional Training

To ensure a standardized approach to implementation of the self-management program, both regionally and internationally, a specific mandatory training workshop was developed for all health care professionals interested in implementing the iFightDepression tool and in guiding patients. The training workshop is 3 hours in length and focuses on the symptomatology and treatment of depression, the concepts of self-management and cCBT, the contents of the iFightDepression tool, and the specific protocols for implementing and guiding the tool in routine practice in addition to assessing individuals for eligibility to use the tool. The development and inclusion of such standardized professional training sessions is innovative as the systematic review informing PREDI-NU indicated that the majority of existing guided cCBT studies do not specify whether the professionals providing guidance and support were specifically trained to use the interventions with clients in a standardized manner. Furthermore, it facilitates the potential for the increased detection and recognition of depression, particularly within primary care services.

#### Evaluation Aims

PREDI-NU primarily focused on assessing the acceptability of the iFightDepression tool and the feasibility of its use for patients, primary care practitioners, and health care professionals. A comprehensive evaluation strategy including quantitative and qualitative analyses of process and outcome measures was integrated throughout all phases of the project. In line with the aim of describing the protocol of the development, implementation, and evaluation of the iFightDepression tool, procedures and instruments of evaluation will be listed briefly below, while a separate future report on the results of the evaluation process will be published after further data have been obtained and analyzed.

Process evaluation comprised focus groups to explore the views, experiences, and recommendations of the professionals guiding the tool, patients using the tool, and healthy Internet users, to obtain more detailed information regarding the acceptability of the tool and the feasibility of its use. Data from the focus groups were transcribed and categorized according to a specific template developed by the PREDI-NU Consortium regarding the iFightDepression tool itself, procedures of implementation and guidance of the tool, recruitment and assessment of patients, and experiences of the professional training.

Outcome evaluation measures included a range of questionnaires developed to assess the specific characteristics of each patient. Baseline measurement of patient characteristics comprised checklists to be completed by both patients and professionals. The professional’s checklist recorded the patient’s mental health history, current treatment, and clinical evaluation. The latter partly drew on the Clinical Global Impression-Severity of Illness measure, which allows for a clinical impression about the current mental health status of the patient to be obtained [56]. The patient’s checklist recorded the patient’s mental health history, their current situation, and attitudes toward and expectations of Web-based self-management. It was also mandatory for patients to complete the PHQ-9 at baseline, 6 weeks, and 3 months after first log-in. The postintervention assessment at 3 months comprised additional items addressing their experience of the iFightDepression tool. The PHQ-9 was available at all times to patients to regularly assess and monitor their mood at a self-chosen frequency (eg, daily or weekly).

Evaluation measures also included a questionnaire for professionals after training. This questionnaire assessed the adequacy, feasibility, and acceptability of the training program and expectations about working with the iFightDepression tool, including procedures of recruitment and guidance. It is intended that the implementation and evaluation of the tool via professionals will allow for linkage of patients’ data to the clinical appraisal of their GP or health care professional. It is expected that the incorporation of quantitative and qualitative data will ensure a more complete picture of the acceptability and feasibility of the tool.

Finally, the intensity of the intervention was derived from recording the number of users of the tool, number of information materials distributed, number of trainings provided, number of professionals attending training, and number of patients invited to participate in the study.

### Results

Targeting mild to moderate depression, the iFightDepression tool is based on cognitive behavioral therapy and addresses behavioral activation (monitoring and planning daily activities), sleep regulation, problem solving, cognitive restructuring (identifying and challenging unhelpful thoughts), mood monitoring, and healthy lifestyle habits. There is also a tailored version of the tool for young people, incorporating less formal language and additional age-appropriate modules on relationships and social anxiety. The tool is accompanied by a 3-hour training intervention for health care professionals, who are guiding the patients while using the tool.

## Discussion

### Effectiveness of Online Interventions

Evidence exists demonstrating the effectiveness of a number of online interventions for depression that are based on the principles of cognitive behavioral therapy. However, only a small number are supported by robust research evidence. Little evidence exists regarding the acceptability of these interventions or the feasibility of their use, for either individuals experiencing depression or health care professionals managing depression in clinical practice. While a comprehensive review of the literature has demonstrated that “guided” online interventions are more effective than nonguided interventions, there is little evidence regarding the optimal length, content, and type of the guidance. In this paper, we have described the protocol for the development, implementation, and evaluation of a new Internet-based guided self-management program—the iFightDepression tool.

### Implementation of the Ifightdepression Tool

The iFightDepression tool can be considered innovative for a number of reasons. It is free of charge for both professionals and patients to use and implemented through health care professionals with defined standards of referral and guidance. It is multilingual, and is currently available in 9 languages—English, German, Spanish, Catalonian, Dutch, Hungarian, Estonian, Italian, and Bulgarian. In addition, it includes youth-focused modules and a specific module addressing the relationship between sleep patterns and mood. Implementation of the tool was undertaken in a standardized manner, with the development of a specific training workshop for professionals. Finally, the iFightDepression tool was enhanced based on results from an evaluation process that focused on assessing the acceptability of the tool and feasibility of its use, both with patients and health care professionals. The iFightDepression tool therefore represents an evidence-informed and standardized online intervention for individuals with mild to moderate depression that can be implemented throughout Europe in a uniform manner. It is intended that the iFightDepression tool will empower patients by virtue of its focus on increasing the capacity of individuals to self-manage their symptoms of depression with guidance from their health care professional. It is also intended to afford health care professionals a free evidence-based resource for effectively managing depression within their practice in a feasible manner [27], either as an adjunct to treatment as usual or as a single resource where appropriate. As a feasible and evidence-based addition to existing treatment options, the iFightDepression tool and associated professional training represent a promising resource in addressing the growing divide between the number of individuals in Europe who are in need of care as a result of their depression [57], current structural constraints of health systems [58], and the decreasing availability of resources for improving the care of depression. There is the potential for the iFightDepression tool to be used within a stepped-care approach and included in the range of treatment interventions in primary care and mental health services for mild to moderate depression [59]. It is intended that iFightDepression, as a resource that specifically addresses mild to moderate depression, will assist in preventing individuals from developing a more severe form of depression and subsequent suicidal behavior. An additional potential use of the tool could involve that of relapse prevention, as a resource for patients who have recovered from severe depression but who still fall within the mild to moderate range of depression. Furthermore, the iFightDepression tool may be of particular interest and benefit for depressed individuals who may not be able to access face-to-face interventions, such as those with hearing deficits or those who may not be able to travel due to severe chronic physical illnesses.

As the approach was adopted throughout the PREDI-NU project across a number of European regions, it is evident that the iFightDepression materials can easily be transferred to different national and international contexts. Throughout PREDI-NU, several institutions from the project consortium that had not originally planned to implement the iFightDepression tool within their regions have either commenced or planned to commence implementation of the tool, including those in Bulgaria and Belgium. Consequently, there is a clear potential for wider implementation of the tool in other countries and regions.

The funding for the PREDI-NU project ended in August 2014, and the iFightDepression resources are administered via the EAAD, allowing for sustainable use of the iFightDepression tool and the project outcomes. These will be integrated within the materials and procedures of implementation of the 4-level community-based intervention of the EAAD [60] as an addition to the multifaceted approach for improving the care of people with depression and preventing suicidal behavior. In addition, an online version of the standardized training program will be developed and further research will be conducted to evaluate the effectiveness and efficacy of the iFightDepression tool. Thus, there is a clear indication of iFightDepression to complement the range of available resources for mild to moderate depression within primary care and mental health services in Europe.

## Abbreviations

cCBT: Computerized Cognitive Behavioral Therapy
EAAD: European Alliance Against Depression
GPs: general practitioners
OSPI-Europe: Optimising Suicide Prevention Programs and their Implementation in Europe
PHQ-9: Patient Health Questionnaire-9
PREDI-NU: Preventing Depression and Improving Awareness through Networking in the EU
